# *Neocotylidia* gen. nov. (Hymenochaetales, Basidiomycota) Segregated from *Cotylidia* Based on Morphological, Phylogenetic, and Ecological Evidence

**DOI:** 10.3390/jof11050390

**Published:** 2025-05-19

**Authors:** Jinxin Ma, Yizhe Zhang, Jiaqi Liang, Yue Li, Heng Zhao, Zhirui Shang, Jing Si, Haijiao Li

**Affiliations:** 1 State Key Laboratory of Efficient Production of Forest Resources, School of Ecology and Nature Conservation, Beijing Forestry University, Beijing 100083, China; majinxin1999@163.com (J.M.); liyue1218@bjfu.edu.cn (Y.L.); zhaoheng21@bjfu.edu.cn (H.Z.); szhr2003@163.com (Z.S.); 2 National Institute of Occupational Health and Poison Control, Chinese Center for Disease Control and Prevention, Beijing 100050, China; zyz97@263.net (Y.Z.); ljq971202@163.com (J.L.)

**Keywords:** macrofungi, stipitate stereoid fungi, new taxon, taxonomy, phylogeny

## Abstract

Taxonomic and phylogenetic studies of *Cotylidia* (Hymenochaetales, Basidiomycota) were performed. A phylogenetic estimate based on six genetic markers revealed that *Cotylidia* in the current sense includes species belonging to three distantly related clades in the Hymenochaetales. Based on morphology, phylogeny, and ecological habitat, the name *Cotylidia* s.s. is proposed for the first clade, including the type species *C. undulata* and *C. carpatica*. *Neocotylidia* gen. nov. is proposed for the second clade, which includes *N. diaphana*, *N. fibrae*, the new species *N. bambusicola*, and two accessions recorded as *Cotylidia aurantiaca* var. *alba* and *C. aurantiaca*. Contrary to the findings in earlier studies, *C. pannosa* demonstrated a weak grouping affinity with *Globulicium hiemale*, *Hastodontia hastata*, *Atheloderma mirabile*, *Tsugacorticium kenaicum*, *Lawrynomyces capitatus*, and *Lyoathelia laxa*. The morphological characteristics of *Cotylidia* s.s. are restricted to species with hymenial cystidia, pileocystidia, and caulocystidia, as well as a muscicolous habitat. *Neocotylidia* species differ from *Cotylidia* s.s. in the lack of pileocystidia and caulocystidia and substrate preference for soil or wood. Illustrated descriptions of the new species and genus, as well as an identification key to the worldwide species of *Cotylidia* s.l. are provided.

## 1. Introduction

Stipitate stereoid fungi are species with spathulate, or infundibuliform basidiomata, a smooth hymenophore, and smooth, hyaline spores [1]. Previous studies have demonstrated that the stipitate stereoid basidiomata have evolved multiple times and are distributed across several orders of Agaricomycetes, at least including Agaricales Underw., Atheliales Jülich, Hymenochaetales Oberw., Polyporales Gäum., and Stereopsidales Sjökvist, E. Larss., B.E. Pfeil and K.H. Larss. [1,2,3,4,5,6,7,8,9].

As one of the important orders that include corticioid fungi, Hymenochaetales has been continually amended and by now consists of 15 families and 84 genera, among which 19 genera have no definite position at the family level [10,11]. Rickenellaceae Vizzini is a family in Hymenochaetales that includes about 20 genera [12]. Phylogenetic analyses have shown that the family is polyphyletic [1,3,7,8,13,14]. Wang et al. [10] recently amended the description of the family to be monotypic and restricted to the type genus *Rickenella* Raithelh.

*Cotylidia* P. Karst. was previously established under Rickenellaceae by P.A. Karsten [15] and typified through *C. undulata* (Fr.) P. Karst. However, Wang et al. [10] reported that the phylogenetic placement of *Cotylidia* at the family level remains undetermined in Hymenochaetales. Limited phylogenetic studies have grouped *Cotylidia* with species from Resiniciaceae L.W. Zhou and Xue W. Wang, Rickenellaceae, Peniophorellaceae L.W. Zhou, Xue W. Wang and S.L. Liu, and several allied genera that have no definite positions at the family level [8,9,10,11,16,17]. Only five species and one variety of *Cotylidia* have been sampled in previous studies and have never been found to constitute a monophyletic lineage in any monophyletic analysis [8,9]. Thus, further phylogenetic analyses and species collection are needed to clarify the relationships among *Cotylidia* and related genera.

*Cotylidia* species are characterized by infundibuliform or spathulate stipitate basidiomata; smooth or rugose hymenophore; a monomitic hyphal system without clamp connections; hyaline, smooth, inamyloid basidiospores; long protruding hymenial cystidia; and a muscicolous, terricolous, or lignicolous habitat [9,18,19,20,21,22]. Currently, approximately 11 taxa have been classified under *Cotylidia* [9,10,20,22,23,24,25,26,27,28], which includes seven species that were discovered in China [9,18,19,29,30].

During studies on macrofungi in Southwest China, a white, stipitate stereoid material/specimen was collected from the ground under bamboo in the Guizhou and Sichuan Provinces. Further studies showed that it belongs to *Cotylidia* s.l.; however, it could not be classified as any present species. The aim of the work has been to determine the identity of the material found, as well as to clarify the taxonomy and phylogeny of *Cotylidia* species based on morphological evaluation, phylogenetic analysis, and ecological data.

## 2. Materials and Methods

### 2.1. Site Description

The type specimen of the new species was collected from Qianling Park, Guiyang, Guizhou Province, Southwest China. This region has an annual rainfall of approximately 1129 mm, average temperature of 15.3 °C, and elevation of 1100–1396 m. In total, 128 families, 350 genera, and 476 species of vascular plants were discovered in Qianling Park. Its vegetation is tropical-subtropical forests biome dominated by *Castanopsis eyrei* (Champ. ex Benth.) Tutch., *Quercus glauca* Thunb., *Q. fabri* Hance, *Q. acutissima* Carr., *Pinus massoniana* Lamb., *P. fenzeliana* Hand.-Mzt., *Cunninghamia lanceolata* (Lamb.) Hook., *Platycladus orientalis* (L.) Franco, *Liquidambar formosana* Hance, *Keteleeria davidiana* (Bertr.) Beissn., *Tsuga chinensis* (Franch.) Pritz., *Carpinus pubescens* Burk., *Taxus wallichiana* var. *chinensis* (Pilger) Florin, *Cornus controversa* Hemsley, *Ulmus pumila* L., *Celtis sinensis* Pers., *Betula luminifera* H. Winkl., and bamboo, etc. [31,32].

### 2.2. Morphological Evaluation

All specimens were processed and deposited in the herbarium at the National Institute of Occupational Health and Poison Control, Chinese Center for Disease Control (NIOHP, China CDC). Macromorphological characteristics were investigated based on field notes and color photos of basidiomata. Color codes were verified as proposed by Petersen [33]. Microscopy studies were conducted as described by Kout and Zíbarová [22] and Yang et al. [9]. Sections were studied at up to 1000× magnification using a Nikon E 80i microscope and phase contrast illumination (Nikon, Japan). Detailed descriptions of microscopic structures were worded as in previous studies [34,35,36].

### 2.3. DNA Extraction and Sequencing

To obtain polymerase chain reaction (PCR) products from six dried specimens of the new species and *Cotylidia fibrae*, the Phire^®^ Plant Direct PCR Kit (Finnzymes Oy, Espoo, Finland) was used according to the manufacturer’s instructions with some modifications [36]. The ITS1-5.8S-ITS2 region (nuclear ribosomal internal transcribed spacer, ITS), nuclear ribosomal large subunit (nrLSU), nuclear ribosomal small subunit (nrSSU), mitochondrial small subunit (mtSSU), RNA polymerase II second largest subunit (*rpb2*), and translation elongation factor 1α (*tef1α*) regions were amplified using the selected primer pairs ITS5/ITS4 [37], LR0R/LR5 [38], PNS1/NS41 [39], MS1/MS2 [37], bRPB2-6F/bRPB2-7.1R [40], and EF1-983F/EF1-1953R [41]. PCR was performed as described by Tang et al. [36]. All newly generated sequences in this study were deposited in GenBank and are listed in bold in Table S1.

### 2.4. Phylogenetic Analyses

In addition to the newly generated sequences for this study, additional related sequences, mainly based on the work of Yang et al. [9], Wang et al. [10], and Wang and Zhou [11], were also integrated in phylogenetic analyses (Table S1).

All sequences were aligned using ClustalX v.1.83 [42], manually optimized in BioEdit v.7.0.5.3 [43], and compiled in one concatenated dataset for ITS1-5.8S-ITS2, nrLSU, nrSSU, mtSSU, *rpb2*, and *tef1α* markers to explore the phylogenetic position of the newly sequenced specimens in Hymenochaetales. Following the work of Wang and Zhou [11], two species from Polyporales, viz. *Fomitopsis pinicola* and *Grifola frondosa*, were also included, and two species from Thelephorales, viz. *Boletopsis leucomelaena* and *Thelephora ganbajun*, were selected as outgroups. The final alignments and the topologies were deposited in TreeBase (http://treebase.org/treebase-web/home.html, submission ID: 31700, accessed on 14 September 2024).

Maximum likelihood (ML) analyses and Bayesian inference (BI) were carried out using RAxML v.8.2.10 [44] and MrBayes v.3.2.6 [45], respectively. In ML analyses, statistical support values were obtained using rapid bootstrapping with 1000 replicates, with default settings used for the other parameters. For BI analyses, the best-fit models for nucleotide substitution were estimated using jModeltest v.2.17 [46]. Four Markov chains were run for 2,000,000 generations until the split deviation frequency values were lower than 0.01. Trees were sampled every 100th generation. The first quarter of the sampled trees, which represented the burn-in phase of the analyses, was discarded, whereas the remaining ones were used to calculate the Bayesian posterior probabilities (BPPs) in the majority rule consensus.

Phylogenetic trees were visualized using TreeView [47]. Branches that received bootstrap supports for ML (≥50%) and BPPs (≥0.95) were considered significantly supported.

Genetic distances of nrLSU sequences of species belonging to different genera within Hymenochaetales were calculated with the Kimura 2-parameter (K2P) model using MEGA v.11 software [48,49].

## 3. Results

### 3.1. Phylogeny

**Figure 1:**
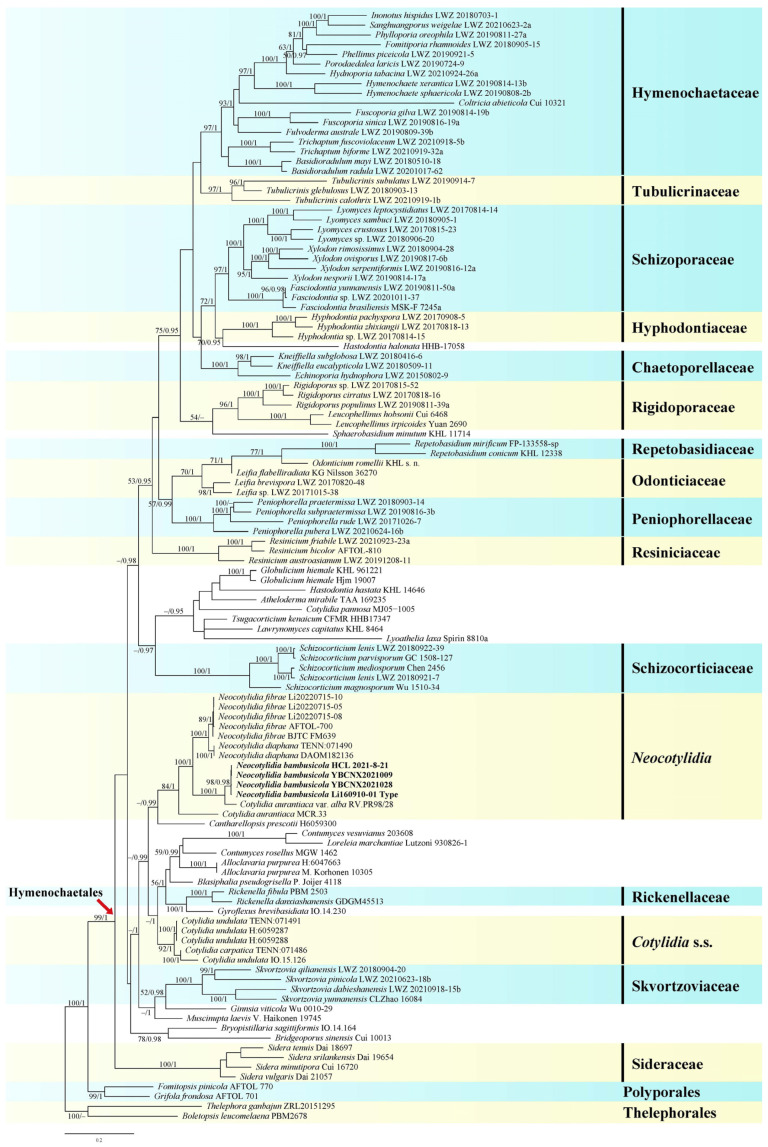
Phylogeny of *Cotylidia* s.l. as determined using maximum likelihood (ML) analyses based on internal transcribed spacer (ITS), nuclear ribosomal large subunit (nrLSU), nuclear ribosomal small subunit (nrSSU), mitochondrial small subunit (mtSSU), RNA polymerase II second largest subunit (*rpb2*), and translation elongation factor 1α (*tef1α*) sequences. Branches are labeled with an ML bootstrap value of >50% and Bayesian posterior probability (BPP) of >0.90. New species are indicated in bold.

The concatenated ITS1-5.8S-ITS2-nrLSU-nrSSU-mtSSU-*rpb2*-*tef1α* dataset contained 107 ITS, 111 nrLSU, 66 nrSSU, 62 mtSSU, 71 *rpb2*, and 45 *tef1α* sequences from 114 samples, representing 101 ingroup taxa and the outgroup (Table S1), and had an aligned length of 5579 characters. jModelTest suggested SYM+I+G, SYM+G, GTR+G, GTR+I+G, GTR+I+G, GTR+G, GTR+I+G, and GTR+I+G to be the best-fit models of nucleotide evolution for ITS1, 5.8S, ITS2, nrLSU, nrSSU, mtSSU, *rpb2*, and *tef1α* markers, respectively, for the Bayesian analysis. The average standard deviation of split frequencies of BI was 0.009318 at the end of the run. BI analyses yielded nearly identical tree topologies with the ML analyses. Only the ML tree is provided in Figure 1 with the likelihood bootstrap values and BPPs labelled along the branches.

*Cotylidia* species were recovered in three distinct clades (Figure 1), namely the *Cotylidia* s.s. clade including the type species *C. undulata* and *C. carpatica* (ML = 92%, BI = 1); the new genus *Neocotylidia* clade including *N. diaphana*, *N. fibrae*, the new species *N. bambusicola*, and two accessions recorded as *Cotylidia aurantiaca* var. *alba* and *C. aurantiaca*. (ML = 84%, BI = 1); and *C. pannosa* (Sowerby) D.A. Reid weakly grouped with *Globulicium hiemale* (Laurila) Hjortstam, *Hastodontia hastata* (Litsch.) Hjortstam and Ryvarden, *Atheloderma mirabile* Parmasto, *Tsugacorticium kenaicum* Nakasone and Burds., *Lawrynomyces capitatus* (J. Erikss. and Å. Strid) Karasiński, and *Lyoathelia laxa* (Burt) Hjortstam and Ryvarden.

The pairwise genetic distances between the clade of *Neocotylidia* and other known genera in the order Hymenochaetales ranged from 0.0422 to 0.1551, which were not lower than the pairwise distance (from 0.0207 to 0.1994) of other genera in Hymenochaetales (Table S2). Hence, we feel that the clade of *Neocotylidia* can be phylogenetically treated as an independent genus in Hymenochaetales.

### 3.2. Taxonomy

***Neocotylidia*** Jing Si and Hai J. Li, gen. nov.

MycoBank No. 850556.

Etymology—*Neocotylidia*: refers to the resemblance to *Cotylidia*.

Type species—*Neocotylidia bambusicola* Jing Si and Hai J. Li.

Description—Basidiomata annual, solitary or gregarious, sometimes confluent, infundibuliform, flabelliform or spathulate, stipitate, coriaceous, usually papery-thin. Pileal surface from white or cream to alutaceous brown, zonate or not, sometimes darker when dry. Hymenophore smooth or rugose, similar to pileal surface color. Stipe central, eccentric to lateral, usually tomentose, floccose or pubescent. Hyphal system monomitic, hyphae simple septate. Hymenial cystidia of tramal origin, hyaline, thin- to thick-walled, aseptate or septate, the cystidia are long and projecting beyond the basidia. Pileocystidia and caulocystidia absent. Basidiospores hyaline, thin-walled, smooth, mostly more or less ellipsoid, rarely cylindrical, clavate to ovate, with or without guttules, IKI−, CB−. Terricolous or lignicolous habitat.

Remarks—Compared with *Cotylidia*, *Neocotylidia* species have no pileocystidia and caulocystidia and grow on soil or wood. The two species subsumed under *Cotylidia* s.s. (*C. undulata* and *C. carpatica*) grow on moss [22,50]. In particular, some fungal anatomical structures in *Mnium thalli* indicate biotrophic parasitism in connection with *C. carpatica* [50].

**Figure 2:**
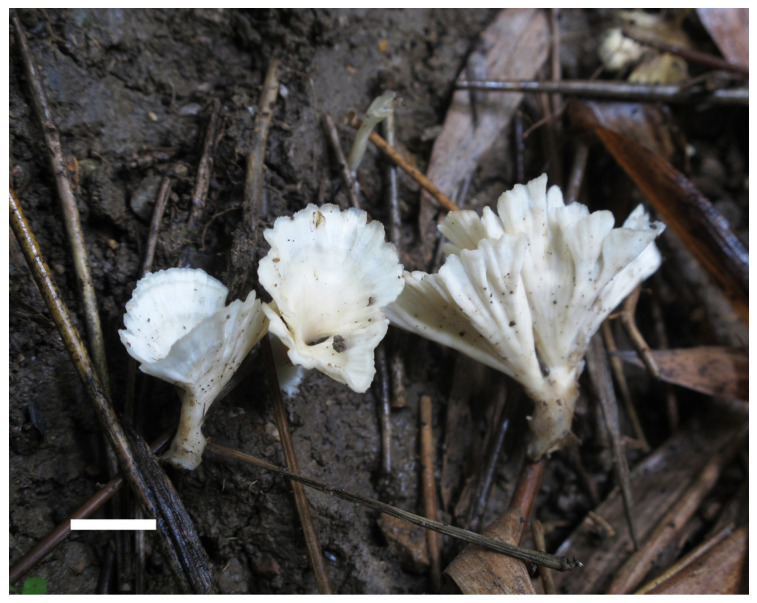
Basidiomata of *Neocotylidia bambusicola* (holotype; bar = 1 cm).

**Figure 3:**
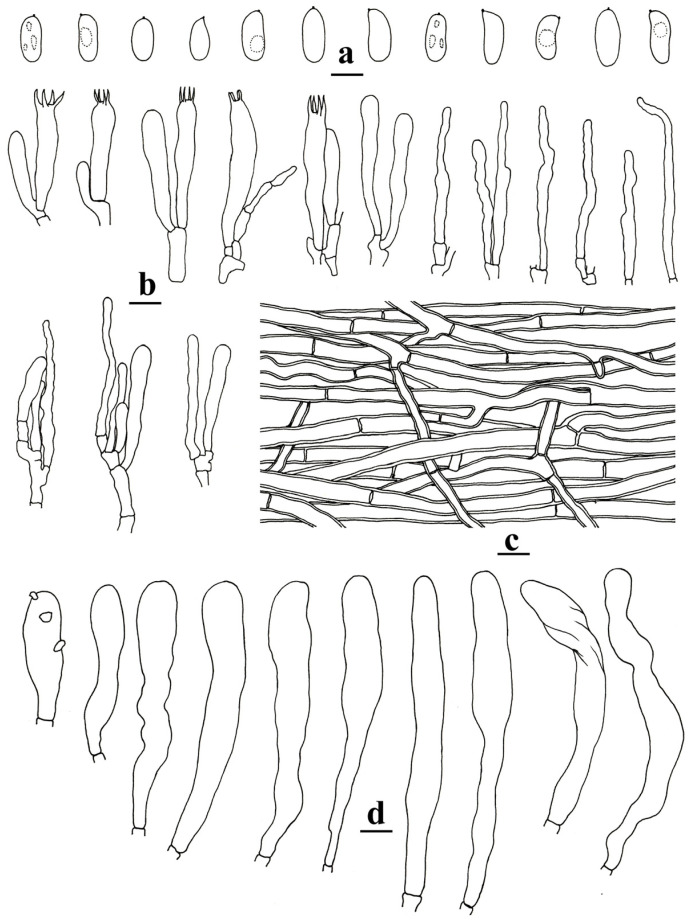
Microscopic structures of *Neocotylidia bambusicola* (*160910-01*, holotype). (**a**) Basidiospores; (**b**) basidia, basidioles, and hyphal ends; (**c**) hyphae from context; (**d**) hymenial cystidia. (bars: (**a**) = 5 µm; (**b**–**d**) = 10 µm). Illustrated by Hai-Jiao Li.

***Neocotylidia bambusicola*** Jing Si and Hai J. Li, sp. nov. (Figure 2 and Figure 3)

MycoBank No. 850559.

Etymology—*bambusicola* (Lat.): refers to this species that grows under bamboo.

Diagnosis—*Neocotylidia bambusicola* is characterized by stipitate basidiomata; infundibuliform, flabelliform to spathulate pilei; glabrous, radially furrowed, white, cream to pale flesh pink pileal surface; tomentose stipe; thick-walled, cylindrical to clavate hymenial cystidia; lack of pileocystidia and caulocystidia; hyaline, cylindrical, clavate to oblong ellipsoid, thin-walled basidiospores, 7–9.3 × 3.2–4.4 µm.

Habitat and distribution—Terricolous under bamboo, at present only reported in Southwest China, summer to autumn.

Type—CHINA. Guizhou Province, Guiyang, Qianling Park, altitude: 1145 m, 26°36′22.36″ N, 106°41′52.33″ E, on the ground under bamboo, 10 September 2016, *Li160910-01* (holotype, deposited at the NIOHP). GenBank accession numbers: ITS OQ376551, nrLSU OQ372914, *rpb2* PP764668, and *tef1α* PP764673.

Description—Basidiomata gregarious, annual, fasciculate, stipitate, coriaceous when fresh and hard, corky upon drying, without odor or taste. Pilei infundibuliform, flabelliform to spathulate, very thin, up to 3.5-cm high and 3-cm wide. Pileal surface glabrous, concentrically and radially zonate, white, cream, and pale flesh pink toward the center. Stipe surface tomentose, white to cream, up to 2 cm in length and 5 mm in diameter. Hymenophore glabrous, white, cream, and pale flesh pink toward the stipe. Flesh under hymenophore very thin, usually less than 1-mm thick, coriaceous.

Hyphal structure—Hyphal system monomitic; all hyphae without clamps, hyphae IKI−, CB−, tissue unchanged in KOH. Generative hyphae in the pileal context hyaline, thick-walled with a wide lumen, branched, more or less parallel along the pileal surface, 4–7 μm in diameter; generative hyphae in the stipe trama similar to those in the pileal context, 3–7.5 μm in diameter.

Microstructure—Hymenial cystidia of tramal origin, hyaline, thick-walled, aseptate, more or less cylindrical to clavate, sometimes encrusted with irregular crystals, remarkably projecting above the hymenium up to 55 μm, 77–104 × 8–11 μm. Pileocystidia and caulocystidia absent. Basidia clavate, with four sterigmata, 30–47 × 6–8 µm; basidioles shaped similarly to basidia but slightly smaller; hyphal ends in the hymenium usually with narrow tips, sometimes with one or two secondary septa. Basidiospores hyaline, cylindrical, clavate to oblong ellipsoid, thin-walled, smooth, sometimes with one to several guttules, IKI−, CB−, [80/4/2] (6.9–)7–9.3(–10.1) × (3–)3.2–4.4(–4.6) µm; L = 8.02 µm; W = 3.7 µm; Q = (1.6–)1.85–2.66(–3); Q_m_ = 2.19 ± 0.25.

Additional specimens examined (paratypes)—CHINA. Guizhou Province, Guiyang, Qianling Park, altitude: 1145 m, 26°36′22.36″ N, 106°41′52.33″ E, on the ground under bamboo, 21 September 2014, *Li 140921-03*; Sichuan Province, Yibin, Changning County, Huatan Town, Shiliang Village, altitude: 378 m, 28°28′18″ N, 104°48′16″ E, on the ground under bamboo, 20 July 2021, *YBCNX2021009*; Meitong Town, Yihong Village, altitude: 528 m, 28°18′25″ N, 105°0′42″ E, on the ground under bamboo, 21 July 2021, *YBCNX2021028*.

Remarks—*Neocotylidia fibrae* (comb. nov. in the present paper), a new species first discovered in China [9] is similar to *N. bambusicola* by its more or less white basidiomata. However, *N. fibrae* has a hymenophore covered with distinct white fibres, wider hymenial cystidia (70–115 × 12.5–20 µm), and smaller basidiospores (5–5.5 × 3–3.5 µm), grows on the ground under *Pinus tabuliformis* Carr., and is distributed in temperate regions in China [9].

*Cotylidia aurantiaca* var. *alba* also forms white, papyraceous basidiomata and forms a clade with the new species *N. bambusicola* (Figure 1). However, *C. aurantiaca* var. *alba* has septate hymenial cystidia with 1–3 transverse septa and ellipsoid basidiospores, usually grows on trunks or woody debris, and was first described in Argentina [18].

*Cotylidia diaphana*, which is combined with *N. diaphana* in a forthcoming manuscript, has more or less white basidiomata similar to those of *N. bambusicola*; however, *N. diaphana* has hymenial cystidia with one septum and smaller, ellipsoid basidiospores (4–6 × 2.75–3.5 µm), and grows on the ground in damp coniferous or frondose woods, or on lawns under trees [24].

*Cotylidia harmandii* was discovered in Japan and is similar to *N. bambusicola* in its creamy to beige pileus, protruding hymenial cystidia, lack of pileocystidia and caulocystidia, and growth on the ground. However, *C. harmandii* differs from *N. bambusicola* by its pinkish or purplish-red pileus when dry, shorter hymenial cystidia (up to 78 µm long), and smaller basidiospores (6 × 3 µm) [18].

Morphologically, *Cotylidia komabensis* is in good accordance with the definition of *Neocotylidia* and has characteristics similar to those of *N. bambusicola*, such as stipitate, white basidiomata, hymenial cystidia, lack of pileocystidia and caulocystidia, and growth on the ground. The type material of *Stereum albidum* Lloyd (1916), a synonym of *C. komabensis*, was found in association with bamboo roots [18]. However, *C. komabensis* differs from *N. bambusicola* by its thin-walled hymenial cystidia and smaller basidiospores (6–8.5 × 2.5–3.5 µm) [18].

*Cotylidia marsicana* is similar to *N. bambusicola* in terms of stipitate, white basidiomata, but it differs from *N. bambusicola* by its distinctly smaller hymenial cystidia (40–60 × 4–6 μm), basidia (15–20 × 4–5 µm), basidiospores (4–5 × 3–3.5 µm), and growth on burnt wood [19].

*Cotylidia pannosa* differs from *N. bambusicola* by its bright red-orange pileal surface margin (all basidiomata when young), larger hymenial cystidia (70–160 × 10–14 μm), wider ellipsoid basidiospores (8–10 × 4–4.6 μm), and growth on bare soil or litter from deciduous trees [22].

***Neocotylidia diaphana*** (Cooke) Jing Si and Hai J. Li, comb. nov.

MycoBank No. 850561.

Basionym—*Stereum diaphanum* Cooke, Syll. fung. (Abellini) 6: 558. 1888.

=*Cotylidia diaphana* (Cooke) Lentz, Agric. Monogr. U.S.D.A. 24: 12. 1955.

Remarks—This species is characterized by stipitate basidiomata; thin coriaceous, normally discrete, and frequently infundibuliform but sometimes pseudo-infundibuliform or even spathulate pileus, which is white or pallid when fresh, becoming creamy-buff, pale ochraceous or straw-colored with a slight sheen when dry; protruding thin- to slightly thick-walled hymenial cystidia that sometimes has 1–3 transverse septa; only hymenial cystidia appearing on the hymenium; ellipsoid, thin-walled basidiospores; and growth on the ground or on the wood of hardwood trees [20]. Phylogenetically, this species is grouped in the clade we have defined as *Neocotylidia* (Figure 1).

***Neocotylidia fibrae*** (L. Fan and C. Yang) Jing Si and Hai J. Li, comb. nov.

MycoBank No. 850562.

Basionym—*Cotylidia fibrae* L. Fan and C. Yang, Phytotaxa. 487(2): 7. 2021.

Remarks—This species was discovered in a temperate region in China in 2021 [9]. It is characterized by more or less white basidiomata with a hymenophore covered with distinct white fibres; protruding hymenial cystidia; lack of pileocystidia and caulocystidia; ellipsoid to oblong, thin-walled basidiospores; and growth on the ground under *Pinus tabuliformis* [9]. Based on the morphological characteristics and phylogenetic analyses (Figure 1) [9], we propose the classification of this species as *Neocotylidia fibrae*.

***Cotylidia aurantiaca*** (Pat.) A.L. Welden, Lloydia 21: 40. 1958.

Basionym—*Podoscypha aurantiaca* Pat., Enum. Champ. Guadeloupe (Lons-le-Saunier): 20. 1903.

Remarks—This species is characterized by stipitate, paper-thin, basidiomata; bright yellow pileus when fresh with fimbriate margin; protruding cylindrical, clavate or slightly capitate to subglobose or pyriform hymenial cystidia; lack of pileocystidia and caulocystidia; thin-walled, ellipsoid basidiospores, and growth primarily on woody substrates, but it may also be terrestrial [18,20], These characteristics indicate that this species may be a *Neocotylidia* species. The type locality of *C. aurantiaca* is in Rio de Janeiro, Brazil. No sequences from the type specimen or the type locality were obtained. Only one sequence (AF261460) from the USA was available at present (Figure 1). However, the sequences (AF261460) from the USA may not be the real *C. aurantiaca*, and further studies are needed to obtain sequences from the type specimen of *C. aurantiaca* or at least from the type locality before a final decision on *C. aurantiaca*.

***Cotylidia aurantiaca* var. *alba*** D.A. Reid, Beih. Nova Hedwig. 18: 67. 1965.

Remarks—One sequences (AF261458) recorded as *Cotylidia aurantiaca* var. *alba* from Puerto Rico was used in our phylogenetic analyses, which strongly grouped with the new species *Neocotylidia bambusicola* (ML = 100%, BI = 1), indicating that this specimen (RV.PR98/28) was undoubtedly a *Neocotylidia* species. Phylogenetic results (Figure 1) also showed that the two sequences recorded as *C. aurantiaca* and *C. aurantiaca* var. *alba* represent two independent species. The type locality of *C. aurantiaca* var. *alba* is from Argentina, and thus the sequences (AF261458) are questionable. Further efforts are needed to acquire sequences from the type specimen or at least other specimens from the type locality to determine its final classification status.

**Notes on other species of *Cotylidia* recorded without molecular data**
***Cotylidia guttulata*** L. Rémy, Bull. trimest. Soc. mycol. Fr. 80(4): 579. 1965.

Remarks—Morphologically, this species is very similar in features to *C. muscigena* except for its biguttulate spores. However, the number of guttules in each spore may vary. Jülich [51] synonymized *C. guttulata* with *C. muscigena*, whereas Moreau et al. [52] consider *C. guttulata* as a form of *C. muscigena* f. *guttulata* (Rémy) P.-A. Moreau, Wuilb. and Courtec. The muscicolous habitat preference of *C. guttulata* and the presence of hymenial cystidia, pileocystidia, and caulocystidia [18] indicate that *C. guttulata* is a *Cotylidia* s.s. species.

***Cotylidia harmandii*** (Lloyd) D.A. Reid, Beih. Nova Hedwigia. 18: 76. 1965.

Basionym—*Stereum harmandii* Lloyd, Mycol. Writ. (Cincinnati) 4(46): 22. 1913.

Remarks—This Japanese species produces stipitate basidiomata; thin, translucent, flabelliform pileus creamy to beige in color, without zonation, and split into several segments; protruding abundant hymenial cystidia (up to 78 × 7–9 μm) which are long, thin-walled, and non-septate; lacks pileocystidia and caulocystidia; and grows on the ground [18]. These distinct features suggest this species belongs to *Neocotylidia*.

***Cotylidia komabensis*** (Henn.) D.A. Reid, Beih. Nova Hedwigia. 18: 77. 1965.

Basionym—*Thelephora komabensis* Henn., Bot. Jb. 31: 736. 1902.

Remarks—This species is characterized by stipitate, white basidiomata; thin, mostly translucent, spathulate, or reniform pileus with a crenulate, incised, or entire margin; long cylindrical, thin-walled, non-septate hymenial cystidia (up to 104 × 9–11 μm); lack of pileocystidia and caulocystidia; elliptical basidiospores (6–8.5 × 2.5–3.5 μm); and growth on the ground in large groups [18], which indicate that it may be a *Neocotylidia* species.

***Cotylidia marsicana*** Lonati, Micol. Veg. Medit. 15(1): 3. 2000.

Remarks—This Mediterranean species is characterized by small, stipitate, white basidiomata; infundibuliform pileus (5–15 × 3–8 mm); white, pruinose stipe (3–5 × 1–2 mm); small, thin-walled, ellipsoid to broadly ellipsoid basidiospores (4–5 × 3–3.5 μm); cylindrical hymenial cystidia (40–60 × 4–6 μm); lack of pileocystidia and caulocystidia; and growth on burnt wood [19], most of which indicate that it may be a *Neocotylidia* species.

***Cotylidia muscigena*** L. Rémy, Bull. trimest. Soc. mycol. Fr. 80(4): 579. 1965.

Remarks—This species is characterized by small, stipitate basidiomata; very thin, semi-translucent, flabelliform to semi-infundibuliform pileus, which is often longitudinally incised; pale yellowish to brownish, sometimes zonate pileal surface, which is finely pruinose; ellipsoid, hyaline, smooth, inamyloid basidiospores (6–8 × 2.5–2.8 μm), protruding aseptate, cylindrical hymenial cystidia (43–70 × 6–10 μm); pileocystidia (40 × 7–9 μm) and caulocystidia (40–70 × 6–10 μm), and growth on living mosses (*Brachythecium*, *Bryum*, and *Mnium*) [18,22]. These features indicate that this species is a *Cotylidia* s.s. species.

***Cotylidia pannosa*** (Sowerby) D.A. Reid, Beih. Nova Hedwigia 18: 81. 1965.

Basionym—*Helvella pannosa* Sowerby, Col. fig. Engl. Fung. Mushr. (London) 2(14): 155. 1799.

Remarks—This species is characterized by stipitate basidiomata, which in groups often become confluent; bright red-orange pileal surface at the margin, ochre toward the center, and changes from rose to cream; large, aseptate hymenial cystidia (70–160 × 10–14 μm); lack of pileocystidia and caulocystidia; large ellipsoid basidiospores (8–10 × 4–4.6 μm), and growth on bare soil or litter from deciduous trees [22]. These characteristics indicate that this species may be a *Neocotylidia* species. A previous phylogenetic analysis based on ITS sequences showed that *C. pannosa* is grouped with *N. fibrae* but is weakly supported by the analyses (ML = 56%, BI < 0.90) [9]. However, *C. pannosa* is weakly grouped with *Globulicium hiemale*, *Hastodontia hastata*, *Atheloderma mirabile*, *Tsugacorticium kenaicum*, *Lawrynomyces capitatu*, and *Lyoathelia laxa* (Figure 1) in the present study.

## 4. Discussion

Our phylogenetic analyses revealed that *Cotylidia* species in the current sense are classified into three distantly related clades, confirming that *Cotylidia* is not monophyletic, and the three clades form genus-level lineages in Hymenochaetales [8,9,10,11,16]. One clade included the type species *C. undulata* and *C. carpatica* (Figure 1), which produce stipitate, coriaceous basidiomata with hymenial cystidia, pileocystidia, and caulocystidia. These basidiomata grow on mosses [9,18,22,52]. The second clade *Neocotylidia* is composed of *N. diaphana*, *N. fibrae,* and the new species *N. bambusicola*, and two samples/specimens recorded as *Cotylidia aurantiaca* var. *alba* and *C. aurantiaca* (Figure 1). All of them grow on soil or wood, only produce hymenial cystidia, and lack pileocystidia and caulocystidia [9,18,22,52]. Unlike the classification reported in a previous study of Yang et al. [9], *Cotylidia pannosa* is weakly grouped with *Globulicium hiemale*, *Hastodontia hastata*, *Atheloderma mirabile*, *Tsugacorticium kenaicum*, *Lawrynomyces capitatus*, and *Lyoathelia laxa* (Figure 1).

*Cotylidia* was previously established under Rickenellaceae [12,16]. Wang et al. [10] and Wang and Zhou [11] reported that the phylogenetic placement of *Cotylidia* (the specimen AFTOL-700 labelled as *Cotylidia* sp.) was also used in the present study, which found it to belong to *Neocotylidia*, at the family level remains undetermined in Hymenochaetales. Phylogenetic analyses have shown that Rickenellaceae is polyphyletic [1,3,7,8,13,14], and it was recently amended to be monotypic and restricted for the type genus *Rickenella* [10].

Limited phylogenetic studies have grouped *Cotylidia* with species from Resiniciaceae, Rickenellaceae, Peniophorellaceae, and several allied genera, *Alloclavaria*, *Atheloderma*, *Blasiphalia*, *Bryopistillaria*, *Cantharellopsis*, *Contumyces*, *Globulicium*, *Gyroflexus*, *Hastodontia*, *Lawrynomyces*, *Loreleia*, *Lyoathelia*, *Muscinupta*, *Sphaerobasidium*, *Tsugacorticium,* and the new genus *Neocotylidia* that have no definite positions at the family level [8,9,10,11,17].

Phylogenetically, *N. bambusicola* is clustered with one specimen *Cotylidia* sp. (HCL 2021-8-21) from China and strongly supported by the phylogenetic analyses (ML = 98%, BI = 0.98; Figure 1); thus, it could be classified as *N. bambusicola*. Current and previous phylogenetic analyses support that the two sequences recorded as *C. aurantiaca* and *C. aurantiaca* var. *alba* (AF261460 and AF261458 for nrLSU) represent two independent *Neocotylidia* species, and these two specimens need further identification [9,18,22,52]. The two sequences were not obtained from the type specimens or specimens from the type localities, which made us doubt whether the identification was accurate. Therefore, before sequences from the type specimens of *C. aurantiaca* and *C. aurantiaca* var. *alba,* or at least sequences from the type locality are available, we would likely retain *C. aurantiaca* and the variety in *Cotylidia*.

No molecular sequences are currently available for *C. guttulata*, *C. harmandii*, *C. komabensis*, *C. marsicana*, and *C. muscigena*; however, based on their morphological characteristics, *C. harmandii*, *C. komabensis*, *C. marsicana*, and *C. pannosa* may be *Neocotylidia* species, whereas *C. muscigena* may belong to *Cotylidia*, and *C. guttulata* may be a form of *C. muscigena* f. *guttulata* belonging to *Cotylidia* [9,18,22,24,52]. *Cotylidia decolorans* was synonymized with *C. aurantiaca* by Ryvarden [20]. Further molecular studies are urgently needed to resolve the taxonomic and phylogenetic locations of these species.
**Key to the worldwide species of *Cotylidia* s.l.**1. Producing hymenial cystidia, pileocystidia, and caulocystidia, growth on moss············21. Only producing hymenial cystidia, growth on soil or wood··············································52. Basidiospores with two guttules································································*Cotylidia guttulata*2. Basidiospores without guttules······························································································33. Basidiospores > 5 µm in length·································································*Cotylidia muscigena*3. Basidiospores < 5 µm in length·······························································································44. Pileus pale alutaceous brown; stipe lateral; basidiospores 3–3.75 × 1.2–2.5 µm·············································································································································*Cotylidia carpatica*4. Pileus buff to brownish, darker in the center; stipe central to eccentric; basidiospores4–5 × 2–2.5 µm···································································································*Cotylidia undulata*5. Basidiomata growing on wood·······························································································65. Basidiomata growing on soil···································································································86. Basidiomata growing on burnt wood, with white basidiomata, small hymenial cystidia(40–60 × 4–6 μm) and basidiospores (4–5 × 3–3.5 µm)································*Cotylidia marsicana*6. Basidiomata most frequently growing on woody substrates, with bright yellow or whitebasidiomata, large hymenial cystidia (up to 122 × 6–26 μm) and basidiospores (6.8–7.5 ×3–3.75 µm)·····································································································································77. Pileus bright yellow··························································*Cotylidia aurantiaca* var. *aurantiaca*7. Pileus white to cream··································································*Cotylidia aurantiaca* var. *alba*8. Hymenial cystidia septate······································································*Neocotylidia diaphana*8. Hymenial cystidia nonseptate·································································································99. Basidiomata more or less white to cream·············································································109. Basidiomata pinkish, purplish-red, or at least bright red-orange at the margin·············1210. Growing on the ground under *Pinus tabuliformis*, hymenophore covered with distinctwhite fibers, hymenial cystidia (70–115 × 12.5–20 µm), basidiospores (5–5.5 × 3–3.5 µm)································································································································*Neocotylidia fibrae*10. Not growing on the ground under *Pinus tabuliformis*, hymenophore without whitefibers··············································································································································1111. Hymenial cystidia thick-walled, basidiospores large (7–9.3 × 3.2–4.4 µm)············································································································································*Neocotylidia bambusicola*11. Hymenial cystidia thin-walled, basidiospores small (6–8.5 × 2.5–3.5 µm)···················································································································································*Cotylidia komabensis*12. Pileal surface bright red-orange at the margin (all basidiomata when young), largehymenial cystidia (70–160 × 10–14 μm) and basidiospores (8–10 × 4–4.6 µm)·······················································································································································*Cotylidia pannosa*12. Pileus of dried material pinkish or purplish-red, small hymenial cystidia (up to 78 ×7–9 μm) and basidiospores (6 × 3 µm)··························································*Cotylidia harmandii*

## Data Availability

All sequence data generated for this study can be accessed via GenBank: https://www.ncbi.nlm.nih.gov/genbank/ (accessed on 15 May 2025).

## Supplementary Materials

The following supporting information can be downloaded at: https://www.mdpi.com/article/10.3390/jof11050390/s1, Table S1: Sources of specimens and GenBank accession numbers for the sequences used in this study. Newly generated sequences are in bold. Table S2: The genetic distances between different genera in the order Hymenochaetales based on nrLSU sequences using the software MEGA v.11. The pairwise genetic distances between the clade of *Neocotylidia* and other known genera are in bold, with the minimum and maximum values on a yellow background. The minimum value and maximum values of pairwise distance of other genera in Hymenochaetales are also in bold with a yellow background.
